# Everybody's Page

**Published:** 1891-10-17

**Authors:** 


### EVERYBODY'S PAGE.

THE *New York Medical Record* quotes an instance of a patient who showed his gratitude to his doctor for curing him of a dangerous disease by giving £100,000, not to the doctor, but to the founding of a hospital. Some people evince their gratitude in a manner more curious than pleasant.

THE number of remedies proposed for the cure of tuberculosis are almost beyond the powers of man to sum up; yet we have tuberculosis in our midst, and to all appearances are likely to find it an unwelcome companion for untold years. Dr. Mannotti has recently been testing the influence of tincture of cloves on the progress of tubercular affections, and has found that it retards considerably the development of the bacillus tuberculosis, and is of most value in cases of cold abscesses. Another cure, or rather prevention, which is much better, has been suggested by someone: "Keep water in the spittoon." This has the merit of simplicity, but everyone does not smoke.

MANY useful discoveries are more or less accidental. This was the case with the discovery of the antiseptic properties of heavy oil of tar. It gradually came into use as a destroyer of vermin on plants and animals; as a wash for sheep it has long been known to all dwellers in the country, yet it is but comparatively few years since it has been introduced into medicine.

IN opposition to Professor Shoemaker, Dr. C. L. Gregory, of California, has great faith in the therapeutic value of "Cactus grandiflorus," holding that it is, beyond any question, one of the most valuable remedies we possess in the treatment of the diseases of the circulatory and nervous systems. His experience has led him to find it especially satisfactory in cases of continued fevers where the pulse was markedly rapid and weak, and in cases of weakened or failing memory depending on nervous debility, and in many cases of cold extremities.

